# Acupuncture for hiccups: Case reports and literature review

**DOI:** 10.1097/MD.0000000000044036

**Published:** 2025-09-19

**Authors:** Yuan Gao, Xiaowei Wang, Xinyi Su, Wenbing Liu, Quanai Zhang

**Affiliations:** a Department of Acupuncture and Moxibustion, Zhejiang Rehabilitation Medical Center, Hangzhou City, Zhejiang Province, China; b Department of Cardiopulmonary Rehabilitation, The Third Affiliated Hospital of Zhejiang Chinese Medical University, Hangzhou City, Zhejiang Province, China; c The Third Clinical Medical College, Zhejiang Chinese Medical University, Hangzhou, China.

**Keywords:** acupuncture, case report, persistent hiccups, TDP, treatment

## Abstract

**Rationale:**

Persistent hiccups following a stroke are a common complication that can adversely affect the patient’s condition and rehabilitation. Certain refractory cases fail to respond adequately to pharmacological treatment. We report 2 cases of successful treatment of persistent hiccups with acupuncture and a medical electromagnetic device (trade name, TDP, an abbreviation of the Chinese phrase “Te-ding Dian-ci-bo Pu”).

**Patient concerns:**

The first patient was a 94-year-old male who had experienced continuous hiccups for 7 days. His comorbidities included Alzheimer disease, cardiac arrhythmia following pacemaker implantation, chronic kidney disease, glaucoma, and recent COVID-19 infection complicated by pneumonia. The second patient was a 70-year-old male who had experienced hiccups for 10 days. He had a history of cerebellar and brainstem infarction, hypertension, and hypopharyngeal carcinoma.

**Diagnoses:**

Both patients were diagnosed with persistent hiccups.

**Interventions:**

Both patients received combined treatment with acupuncture and TDP.

**Outcomes:**

Following treatment, hiccups were alleviated to different degrees, and no recurrence was observed at follow-up.

**Lessons:**

Neuroexcitatory imbalance and thoracoabdominal pressure asymmetry are considered underlying causes of persistent hiccups. Acupuncture combined with TDP may modulate periumbilical arteriovenous networks and abdominal pressure, thereby relieving hiccups. This case series suggests a novel, easily implemented, well-tolerated therapeutic option for the management of persistent hiccups.

## 1. Introduction

Hiccups are a common complication associated with a variety of medical conditions.^[1]^ They are involuntary events characterized by the sudden glottic closure due to spasmodic diaphragmatic contractions. Hiccups lasting more than 48 hours are defined as “persistent hiccups” and can significantly impair quality of life.^[2]^ Although hiccups are often self-limiting, the adverse effects of prolonged persistence should not be overlooked.. Potential complications include insomnia, dysphagia, and aspiration pneumonia, particularly in patients with stroke.^[3]^ Effective treatment is urgently needed.^[4]^

Various therapeutic approaches have been employed to treat hiccups, such as pharmacotherapy and neuromuscular electrical stimulation. However, there are specific adverse effects associated with pharmaceutical therapies, such as agranulocytosis brought on by chlorpromazine,^[5]^ and peripheral edema, drowsiness, and dizziness brought on by gabapentin.^[6]^ In rare cases, neuromuscular electrical stimulation may also cause esophageal damage, complicating the recovery process.^[7]^ It has been demonstrated that acupuncture is an effective way to lessen the frequency and severity of hiccups. However, some acupuncture strategies require patients to maintain a fixed prone position for a long period,^[8–10]^ which can be challenging to implement. Recently, we identified a therapeutic approach of acupuncture combined with a medical electromagnetic instrument (trade name, TDP, an abbreviation of the Chinese phrase “Te-ding Dian-ci-bo Pu”) that yielded satisfactory results in treating persistent hiccups. Herein, we report 2 cases of intractable or persistent hiccups successfully treated using this combined method.

## 2. Case report

### 2.1. Case 1

A 94-year-old male inpatient presented with persistent hiccups lasting 7 days. Seven days earlier, he developed hiccups (approximately 18 hiccups per minute) that failed to resolve spontaneously, following pneumonia secondary to a COVID-19 infection. Administration of metoclopramide injection was ineffective in relieving the hiccups. His medical history included 15 years of hypertension, 10 years of Alzheimer disease, 5 years of cardiac arrhythmia following pacemaker implantation, 2 years of chronic kidney disease and glaucoma. One year earlier, the patient had experienced coughing, expectoration, and dyspnea, accompanied by elevated white blood cell count, decreased oxygen saturation, and impaired sputum clearance. He underwent orotracheal intubation and received mechanical ventilation. During the current hospitalization, following extubation, he experienced chest tightness, dyspnea, confusion, cough, and expectoration. Nasal feeding and high-flow oxygen were administered.

Seven days after COVID-19 infection, the patient’s dyspnea worsened. After diagnostic CT scans and blood tests, anti-infective and expectorant therapies were administered. Meanwhile, hiccups developed. Despite various treatments, the hiccups were not relieved. The hiccups could persist for more than 2 days, worsened at night and accompanied by abdominal undulations. The patient’s family sought acupuncture treatment. Vital Signs and Physical Exam during Hiccups showed: Temperature, 37.6°C; Heart rate, 98 bpm (paced); Respiratory rate, 20 breaths/min; Blood pressure, 148/65 mm Hg. Altered mental status, sluggish pupillary light reflex, blindness, coarse breath sounds bilaterally, normal muscle tone, poor cooperation with strength or sensory testing, positive Babinski sign on the left side. Laboratory tests revealed the following: WBC, 10.05 × 10^9^; RBC, 2.62 × 10^12^; percentage of neutrophil, 86.9%; percentage of lymphocyte, 6.9%; creatinine, 113.7 μmol/L; Na+, 149.4 mmol/L; K+, 5.5 mmol/L; EPI, 47.68%; total protein, 64.4 g/L; albumin, 30.4 g/L; anemia. Imaging findings were as follows: Head CT showed hemorrhage in right basal ganglia. Chest CT showed minor infectious infiltrates bilaterally, pleural effusion bilaterally, status post pacemaker implantation. Abdomen CT showed bilateral renal atrophy and cysts, multiple diverticula of the ascending colon, small hernia at the terminal ileum.

**Figure 1.**
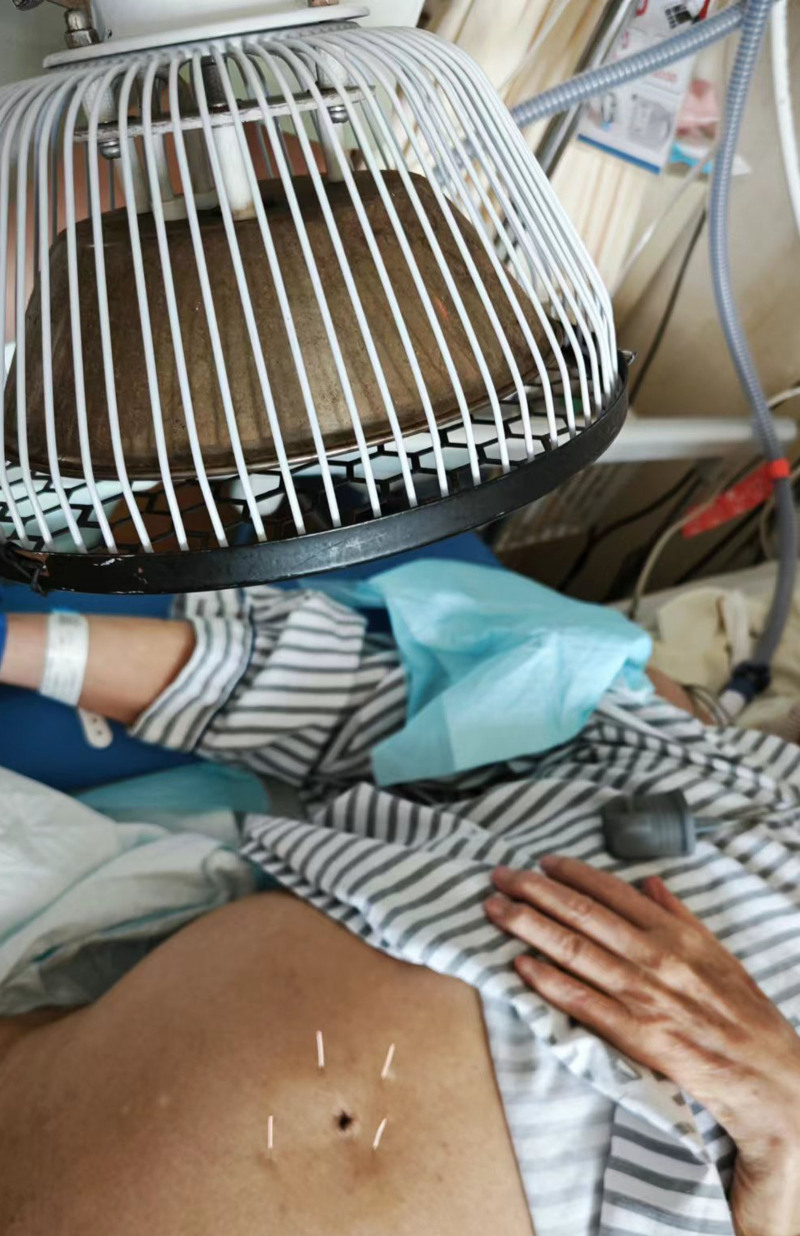
Protocol used for case 1.

Adjustments to the gastric tube length, administration of spasmolytics, and acupuncture at Cuanzhu (BL2) and Hegu (LI4) failed to provide sufficient relief from persistent hiccups during the first week of hospitalization. Therefore, a combined treatment approach was implemented. With the supine position, the periumbilical abdominal skin was exposed. Bilateral Huanghu (KI16), Shuifen (CV9), and Qihai (CV6) were selected. After routine iodine disinfection of the local skin, needles (0.25 mm in diameter, 40 mm in length; Huatuo brand, Suzhou Medical Supplies Factory Co., Ltd., Suzhou, China) were vertically inserted to a depth of about 30 mm at each point, until the acupuncturist felt De Qi (sensation of qi arrival), TDP (Guoren, Chongqing Guoren Medical Equipment Co., Ltd., Chongqing, China) was applied concurrently, with the TDP lamp positioned approximately 0.3 m away from the skin of the needling points in the abdomen for 1 hour (Fig. 1).

Outcomes: Thirty minutes after treatment, the hiccups were gradually resolved. However, intermittent hiccups recurred in the next 3 nights, and 3 additional acupuncture treatments were administered soon after their occurrence respectively. To consolidate the therapeutic effect, the patient underwent 2 courses of treatment (once daily, 5 days a week as 1 course). The frequency, severity, and duration of hiccups were significantly reduced.

### 2.2. Case 2

A 70-year-old male patient was admitted with a 10-day history of intractable hiccups (20 times/min) unresponsive to previous treatments, necessitating further management. One month before admission, the patient suddenly developed limb weakness and loss of consciousness. MRI showed cerebellar and brainstem infarctions. During that time, he experienced vomiting and developed aspiration pneumonia, complicated by dyspnea and copious sputum production, necessitating tracheotomy and mechanical ventilation. He received treatments to improve circulation, lower lipids, and inhibit platelet aggregation, and underwent anti-infective therapy. While ventilator support was later discontinued, the tracheotomy remained in place. He had a history of hypertension for more than 20 years and a hypopharyngeal tumor for 4 years, with ongoing chemotherapy. On admission, vital signs were as follows: temperature, 36.8℃; heart rate, 80 bpm; respiratory rate, 20/min; blood pressure, 130/70 mm Hg; GCS, 4 + T + 3; coarse breath sounds bilaterally, normal muscle tone. However, he did not cooperate with strength or sensory testing and demonstrated bilateral positive Babinski signs. Lab tests revealed the following results: hemogram: WBC, 11.02 × 10^9^; RBC, 3.31 × 10^12^; percentage of neutrophil, 84%; percentage of lymphocyte, 9.2%; CRP, 22.9 ng/L; PCT, 0.65 ng/mL; ALT, 82.4 U/L; total protein, 54.2 g/L; albumin, 27.6 g/L. Head CT imaging showed infarction of the right cerebellar hemisphere and brainstem, partial basilar artery occlusion. Chest CT revealed sputum retention in the left lower lobe bronchi, bilateral pneumonia, and small bilateral pleural effusions.

**Figure 2.**
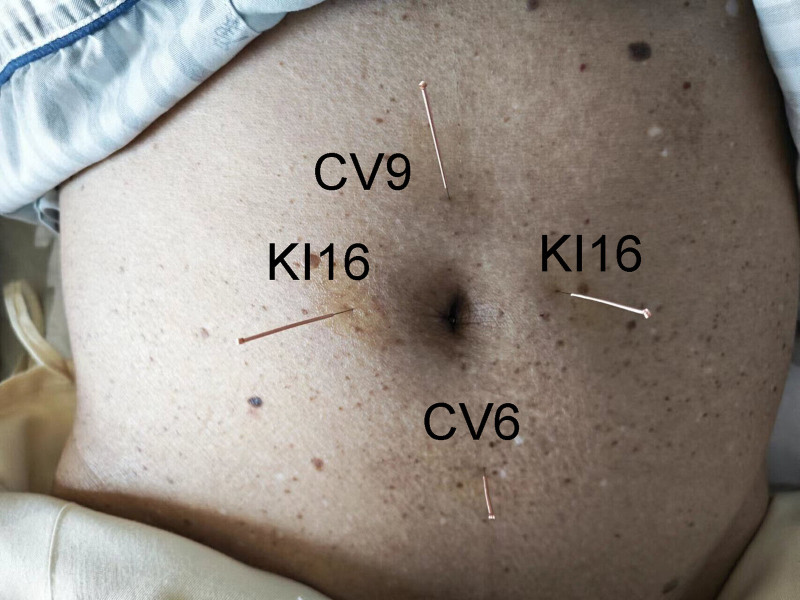
Acupoints used in case 2.

The acupuncture and TDP therapy protocol followed was identical to that in Case 1 (Fig. 2). The patient experienced no hiccups that evening after the first treatment. However, given the history of hypopharyngeal tumor, the hiccups continued, though with a significantly reduced frequency, severity, and duration compared to before. Notably, during subsequent treatments, acupuncture was generally performed in the morning, and intermittent hiccups occurred at night. No hiccups occurred on the night when an additional afternoon acupuncture session was provided, and TDP irradiation of the periumbilical area alone proved ineffective in alleviating the hiccups. Similarly, acupuncture at the 4 spots without TDP also yielded minimal relief of hiccups. Ultimately, it was found that only the fully described protocol successfully resolved the hiccups, which is an intriguing finding. The combined acupuncture and TDP protocol took effect more quickly in this patient. At the 3-month follow-up, there had been no recurrence of persistent or intractable hiccups.

## 3. Literature review

We used the subject terms “acupuncture,” “moxibustion,” or “electroacupuncture” in combination with “intractable hiccups,” “persistent hiccups.” and “case reports.” We searched for relevant reports in PubMed between 2002 and 2023 and reviewed only the full-text articles of case reports in English. Seven studies detailed the patients’ gender, age, primary diagnosis, hiccup duration and frequency, the longest single episode, acupuncture protocols, and recovery outcomes (see Table 1).

**Table 1:** Characteristics of acupuncture for hiccup case reports in literatures.

	Publication year	Authors	Patient age/sex	Primary diease	Hiccups lasting time	Frequence of hiccups/maximum duration	Treatment/lasting time	Regions and acuponits	Hiccup resolution (after treatment)
1	2002	Schiff^[10]^	60/M	Adenocarcinoma of the stomach with liver metastases	4 wk	Non/Non	Acupuncture/20 min	Abdomen: Cv 12 (Zhong wan), midpoint between xiphoid and umbilicus; upper limb: Pc 6 (Nei guan), 2 cm proximal to the wrist crease between palmaris longus and flexor carpi radialis (bilateral); low limb: St 36 (Zu san li), lateral to the tibial tubercle (bilateral); Liv3 (Tai chong), dorsal aspect of foot, between first and second metatarsal-phalangeal joint (bilateral); Sp 4 (Gong sun), medial aspect of foot distal to first metatarsal bone (bilateral); back: Bl-17 (Ge shu), paravertebral points at the level of T6–T7 (bilateral)	The patient stopped hiccuping after an hour, hiccups completely resolved and did not recur during a follow-up period of 1 mo.
			54/M	metastatic lung adenocarcinoma	2 wk	Every 1–2 min and each bout consisted of 4–6 hiccups	Acupuncture/20 min		
2	2005	Liu^[11]^	77/M	Acute Myocardial Infarction	7 d	Non/Non	Acupuncture/10 min	Back: GV14 (Dazhui)	Hiccups ceased after acupuncture. No recurrence of hiccups at the 1-mo follow-up.
3	2006	Lin^[12]^	36/M	Heart and lung transplant recipient	3 d	20 to 25 times per minute.	Acupuncture/2 min	Back: bilateral BL-17 (ge shu), located 1.5 inches lateral to the lower border of the spinous process of the 7th thoracic vertebra	Stopped after 2 min. No recurrence of hiccups for 12 mo
4	2006	Chang^[9]^	67/M	acute cerebral infarction in the right middle cerebral artery	After stroke	5 to 10 s from then on, and prolonged to several hours without termination/non	Near-Infrared Irradiation/3 min each point	Upper Limb : PC 6 (Nei Guan), which is 2 cm proximal to the wrist crease between palmaris longus and flexor carpi radialis; Low Limb: ST 36 (Zu San Li), lateral to the tibial tubercle; Back: DU 9 (Chih Yang), in the interspinal space between T7–T8	ameliorated after 3 h and did not recur for the remainder of the patient’s hospital stay and outpatient follow-up disappeared hours later. No recurrence of hiccups for 1 mo
			74/M	stroke	After stroke	Non/Non			
5	2008	Dietzel^[13]^	Middle-age/M	pancreatic surgery	65 d	10–12 times per minute	Acupuncture/40 min	Auricular points: Diaphragm, Lung, Stomach and Shenmen. GV26 and P6 on both sides	the duration of hiccup phases were continuously reduced and disappeared completely on the postoperative day 72
6	2020	Zhang^[8]^	46/M	Depression	7-yr	Non/3–4 h	Extracranial acupuncture	Neck: 0.5 cm distal to the point of attachment between the occipital ligament and the external occipital protuberance	Resolved completely by the time the patient had completed 3 courses of treatment. No recurrence of hiccups for 1 yr
			75/M	Mallory-Weiss syndrome	7 d	20 per min/1 h			Immediately disappeared. No recurrence of hiccups for 6 mo
7	2023	Huang^[14]^	38/F	Chronic gastritis	1 mo	None/none	Auricular massage, bloodletting at the ear tips, gua sha	Auricular points	Steady improvement

The literature indicates that acupuncture has demonstrated positive therapeutic effects on hiccups occurring in various acute and chronic disease states. These conditions include cancer,^[10]^ post-acute myocardial infarction,^[11]^ post-organ transplantation,^[12]^ cerebrovascular disease,^[9]^ gastrointestinal diseases,^[14,15]^ postoperative states,^[13]^ and emotional disorders.^[15]^ The techniques employed encompass conventional acupuncture,^[10–12]^ acupotomology,^[15]^ near-infrared irradiation,^[9]^ auricular acupuncture,^[13]^ acupressure and massage.^[14]^

Existing reviews have systematically summarized relevant pharmacological and physical therapies for treating hiccups.^[16]^ However, there is currently no literature that elucidates the differences in acupuncture approaches to treating hiccups. Furthermore, acupuncture treatments differ in terms of acupoints and technique selection. Through a review of the literature, we discovered that commonly used acupoints are concentrated in the following regions: abdomen (the surface projection area of the stomach)^[10]^; back (areas where spinal nerves originate from or pass through)^[9,12]^; neck (regions associated with the vagus nerve ganglion)^[11,15]^; ears (the auricular branch of vagus nerve).^[13,14]^ Some reports have also identified acupoints located on the limbs.^[9,10]^ Notably, abdominal acupoints are rarely mentioned.

## 4. Discussion

Hiccups can occur in the context of various diseases, and persistent or intractable hiccups often negatively affect prognosis. As previously stated, pharmacological interventions in Western medicine are often associated with adverse effects. In contrast, acupuncture therapy can lessen the frequency and duration of hiccups. Moreover, most of the methods listed in Table 1 require patients’ cooperation, such as keeping a prone position or holding their breath, which can be challenging for bedridden patients. Our regimen combines acupuncture with TDP therapy and is both simple and easy to administer. It has generated satisfactory results even patients who have been bedridden long-term and are unable to change position independently. Here, we investigate the feasibility of employing this regimen to treat intractable hiccups.

From the traditional perspective, hiccups are considered a protective reflex to prevent excessive breathing, mediated by the efferent motor fibers of the recurrent laryngeal branch of the vagus nerve, which prompts laryngeal closure.^[17]^ Imbalanced thoracoabdominal pressure is a key factor, involving the diaphragm, vagus nerve, sympathetic nerves (T6-T12), periaqueductal gray matter of the brainstem, and basal ganglia involved in information processing, as well as motor nerves innervating the diaphragm and intercostal nerves innervating the intercostal muscles.^[18]^ Excessive abdominal pressure, as opposed to thoracic pressure, may result in hiccups. Similar to hiccups during early pregnancy,^[19]^ hiccups are caused by the elevation and spasms of the diaphragm due to increased abdominal pressure. The Valsalva maneuver utilizes pressure principles: patients inhale and hold their breath, creating positive airway pressure and increasing intrathoracic pressure. This lowers the diaphragm and stimulates the vagus nerve,^[20]^ thereby stopping hiccups. Similarly, some researchers have reported using external methods, such as the Heimlich maneuver,^[21]^ to stop diaphragmatic contractions and relieve hiccups.

**Figure 3.**
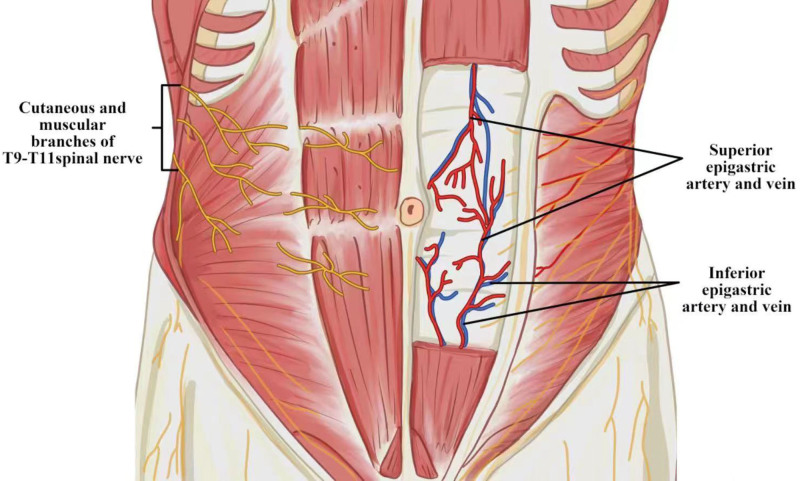
A schematic illustration of the appropriate abdominal wall anatomy for acupuncture therapy of hiccups.

Considering the imbalanced cavity pressure, we make innovative use of uncommon acupoints near the navel. The literature review shows that acupoints in the neck, stomach region, and ears are frequently used. Acupuncture may exert its therapeutic effect by activating nerve circuit.^[22]^ Acupoints CV 6, CV 9, and bilateral KI 16 used in our protocol are close to the navel. Anatomically, the cutaneous and muscular branches of T9-T11 spinal nerves are distributed around these 4 acupoints, and the venous plexus is abundant. On 1 hand, the umbilical cord contains 3 blood vessels - 2 arteries and 1 vein. Twisting of the umbilical vein can obstruct backflow to the inferior vena cava, raise abdominal pressure, and limit diaphragm descent, resulting in infantile hiccups.^[23]^ On the other hand, there is a dense arteriovenous network surrounding the navel. Larger arteriovenous vessels, 1 mm in diameter, are extensively distributed around the navel. The area of greatest density of vertical abdominal wall arteriovenous branches is within 5 cm of the navel,^[24,25]^ while transverse branches are within 2 cm above^[24]^ and 8 cm below the navel.^[26]^ This distribution is relatively consistent with the selected acupoints. Acupoints CV 6, CV 9, and bilateral KI 16 used in our protocol may exert their therapeutic effect by activating the cutaneous and muscular branches of T9-T11 spinal nerves, as well as regulating the blood flow in the rich arteriovenous network around the navel (Fig. 3). According to 1 study,^[27]^ electroacupuncture reduces abdominal pressure while also ameliorating acute gastrointestinal injury secondary to traumatic brain injury. TDP is a commonly used auxiliary physical therapy in the acupuncture department.^[28]^ It generates electromagnetic waves similar to near- and mid-infrared wavelengths of 0.65 to 50 μm.^[29]^ These rays penetrate the skin, promote blood flow,^[30]^ relax intestinal smooth muscle, and facilitate gastric emptying.^[31]^ Thus, acupuncture combined with TDP radiation therapy may effectively treat abdominal pressure, reduce hiccup bouts, and shorten the duration of hiccups. This is a plausible explanation for the effectiveness of acupuncture combined with TDP in treating hiccups.

Our treatment can effectively alleviate persistent hiccups and are well accepted by patients and families. The Valsalva maneuver, often known as breath-holding, is a standard method for regulating thoracoabdominal pressure. Some studies employ laser acupuncture^[9]^ or minimally invasive needle-knife techniques^[15]^ that require the patient to maintain a prone position. For example, the Alzheimer patient and tracheostomy patient in Cases 1 and 2 cannot easily complete the breath-holding maneuver or easily maintain the prone position. Our protocol perfectly addresses these difficulties. For these special patients, acupuncture coupled with TDP requires just the supine position without patients’ cooperation or extra movement, enabling safe operation.

Notably, both cases had nasal tube insertion, which is a cause of abdominal distension.^[32]^ Additionally, previous case reports have described patients’ preference for drinking hot water and avoiding chills.^[15]^ Case 1 also had a history of excessive fluid intake, which may have triggered persistent hiccups. In Case 2, the patient had a history of a malignant tumor in the hypopharynx, which may have contributed to the complexity of the hiccup episodes. During treatment, we found that neither acupuncture alone nor TDP applied to acupoints around the navel effectively relieved hiccups. Only the combination of acupuncture and TDP significantly reduced both the frequency and duration of hiccups. This suggests a complementary effect between acupuncture and TDP. Furthermore, we observed that temporarily adding an extra treatment in the afternoon distinctly alleviated frequent hiccups at night, indicating a specific dose-response relationship for this regimen.

In summary, the onset of hiccups may be attributable to vagus nerve excitation and thoracoabdominal pressure disequilibrium. Based on these 2 cases, acupuncture combined with TDP appears to be a safe, stable, and remarkably effective treatment option for patients with hiccups, particularly those who are bedridden and unable to move independently. Prospective high-quality studies with large sample sizes are needed to confirm its therapeutic advantages.

## Acknowledgments

We thank the patient for consenting to publication of this report.

## Author contributions

**Conceptualization:** Wenbing Liu.

**Data curation:** Yuan Gao.

**Formal analysis:** Xinyi Su.

**Funding acquisition:** Yuan Gao, Quanai Zhang.

**Investigation:** Wenbing Liu.

**Project administration:** Yuan Gao, Xiaowei Wang, Wenbing Liu, Quanai Zhang.

**Resources:** Xiaowei Wang.

**Supervision:** Yuan Gao.

**Writing – original draft:** Yuan Gao, Xinyi Su, Wenbing Liu.

**Writing – review & editing:** Yuan Gao, Xinyi Su, Quanai Zhang.
